# Charcot's triad

**DOI:** 10.1186/1865-1380-4-18

**Published:** 2011-04-27

**Authors:** Jean Louis Frossard, Florent Bonvin

**Affiliations:** 1 Service of Gastroenterology and Hepatology, Geneva University Hospitals, Rue Gabrielle Perret Gentil 24, 1211 Geneva, Switzerland; 2 Department of Radiology, Geneva University Hospitals, Rue Gabrielle Perret Gentil 24, 1211 Geneva, Switzerland

## Abstract

Biliary stones are usually found in the gallbladder, but about 10-20% may spontaneously migrate into the common bile duct where they either remain trapped or migrate subsequently via the papilla of Vater into the duodenal lumen. In some cases, biliary stones may form de novo in the common bile duct because of local precipitating factors. We here present a spectacular case of huge gallstones impacted in the common bile duct (empierrement of the common bile duct) that led to the development of acute cholangitis with septic shock. Urgent nocturnal percutaneous cholangiography permitted biliary drainage and resolution of the cholangitis while the stones were secondarily removed surgically because of the large size of the stones.

Acute suppurative cholangitis may be fatal unless adequate biliary drainage is obtained in a timely manner. The association of fever and rapid onset of jaundice in elderly patients should always make physicians think of cholangitis.

## Introduction

Gallstone disease is one of the most prevalent of all digestive diseases in the United States and Europe. Gallstones do not induce symptoms in the majority of cases, but only 2% to 4% of patients become symptomatic each year [1].

## Case presentation

**Figure 1:**
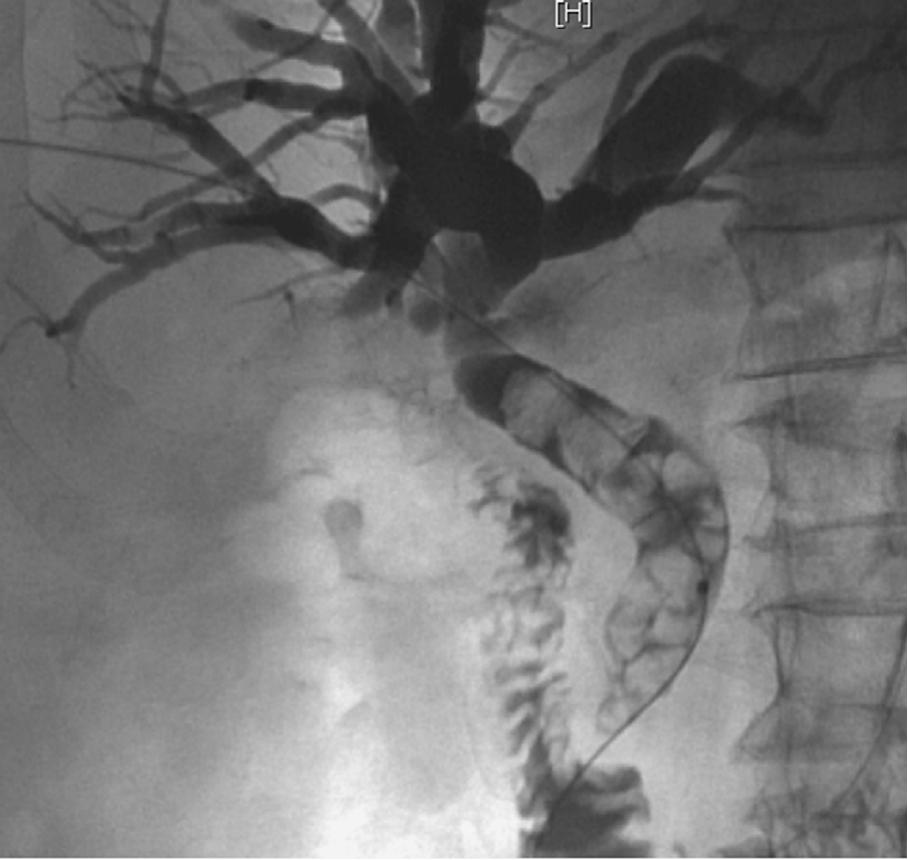
**Percutaneous cholangiography showing a dilated common bile duct containing multiple calcified stones (*arrows*) impacted above the papilla of Vater**.

A 82-year-old man with a previous history of open cholecystectomy performed 12 years ago presented with a three-day history of fever, chills and progressive jaundice. Physical examination showed a temperature of 39.0°C that partially decreased after paracetamol administration. His blood pressure was 85/40 mm Hg, and his pulse rate was 112/min. A complete blood count showed a leukocyte count of 34,100 cells/mm^3^, and the CRP level was 311 mg/dl. His liver function tests revealed a total bilirubin of 210 umol/l, an ALT of 175 U/l and an alkaline phosphatase of 394 U/l. Percutaneous abdominal ultrasound identified a dilatation of both the extrahepatic and intrahepatic biliary tract with high suspicion of stones in the common bile duct. Antibiotics (cefuroxim and metronidazol) were then intravenously administered while waiting for diagnosis confirmation by CT scan the day after admission. Unfortunately, at night the patient developed an acute cholangitis with septic shock that required admission to the intensive care unit and an urgent computed tomography scan. The CT scan showed an enlarged common bile duct and confirmed the presence of several stones impacted in the common bile duct from the papilla to the liver hilum (Figure 1). Because endoscopic drainage was not available at night, a CT scan was followed by percutaneous cholangiography that permitted a temporary drainage; aspiration of pus was sent to the laboratory and confirmed a typical massive "empierrement" of the common bile duct. After 48 h of antibiotics, the patient's condition was stable. Because of the size (>3 cm) of the common bile duct stones, endoscopic removal was not attempted, and the patient was electively scheduled for open choledochotomy and stone removal. Because of technical problems, the planned surgery was not possible, and a biliary-digestive anastomosis was performed instead without any further complications.

## Discussion

Gallstone disease is one of the most prevalent of all digestive diseases in the US and Europe. Gallstones do not induce symptoms in the majority of cases, but only 2% to 4% of patients become symptomatic each year. While the vast majority of patients with gallstone disease should be managed by observation alone, selective cholecystectomy is indicated in defined subgroups of subjects, with an increased risk for the development of gallstone-related symptoms and complications in order to alleviate symptoms of pain, jaundice and to prevent acute pancreatitis, cholangitis and cholecystitis [2]. Common bile duct stones are classified according to their origin: (1) primary bile duct stones, forming initially in the bile duct (i.e., the current case); (2) secondary to gallbladder stones, originating in the gallbladder and passing spontaneously into the bile duct; and (3) secondary to or coexisting with intrahepatic bile duct stones [3]. Patients with infected bile duct stones typically present with fever, abdominal pain and jaundice (Charcot's triad), and in severe cases may also have associated hypotension and mental confusion (Raynold's pentad), which predicts a poor clinical outcome [4]. Precipitating factors of symptomatic stones, such advanced age, comorbid neurological disease and peripapillary diverticulum, were identified as independent risk factors for the development of acute suppurative cholangitis in patients with bile duct stones [5]. In the current case, urgent percutaneous biliary drainage was first performed to stabilize the patient, followed 2 weeks later by biliodigestive anastomosis, as proposed by current available guidelines [6].

## Conclusion

This case illustrates a spectacular impaction of several huge stones in the common bile duct that remained otherwise asymptomatic for years and illustrates the famous Charcot's triad. Acute suppurative cholangitis may be fatal unless adequate biliary drainage is obtained in a timely manner. The major cause of acute suppurative cholangitis is bile duct stones that result either from spontaneous migration from the gallbladder into the common bile duct or appear de novo because of precipitating factors.

## Competing interests

The authors declare that they have no competing interests.

## Authors' contributions

JLF and FB equally analyzed and interpreted the patient data. FB performed the percutaneous biliary drainage.

## Consent

Written informed consent was obtained from the patient for publication of this Case report and any accompanying images. A copy of the written consent is available for review by the Editor-in-Chief of this journal.

## References

[B1] Frossard JL Morel P Detection and management of bile duct stones Gastrointest Endosc 2010 72 808 816 10.1016/j.gie.2010.06.033 20883860

[B2] Frossard JL Steer M Pastor C Acute pancreatitis Lancet 2008 371 143 152 10.1016/S0140-6736(08)60107-5 18191686

[B3] Tazuma S Gallstone disease: Epidemiology, pathogenesis, and classification of biliary stones (common bile duct and intrahepatic) Best Pract Res Clin Gastroenterol 2006 20 1075 1083 10.1016/j.bpg.2006.05.009 17127189

[B4] Agarwal N Chander Sharma B Sarin S Endoscopic management of acute cholangitis in elderly patients World J Gastroenterol 2006 28 6551 6555 10.3748/wjg.v12.i40.6551 PMC4100647 17072990

[B5] Barkun A Barkun J Fried G Ghitulescu G Steinmetz O Pham C Meakins J Goresky C Useful predictors of bile duct stones in patients undergoing laparoscopic cholecystectomy. McGill Gallstone Treatment Group Ann Surg 1994 220 32 39 10.1097/00000658-199407000-00006 7517657 PMC1234284

[B6] Martin D Vernon D Toouli J Surgical versus endoscopic treatment of bile duct stones Cochrane Database Syst Rev 2006 19 CD003327 10.1002/14651858.CD003327.pub2 16625577

